# Supplementary material for: Are carbon emissions trading and green financial instruments synergistic? -Comprehensive quantitative research based on content analysis
Source: PLoS One. 2024 Mar 7;19(3):e0298601. doi: 10.1371/journal.pone.0298601 (PMC10919601; doi:10.1371/journal.pone.0298601)
Supplement: S1 Data — (ZIP) [file pone.0298601.s001.zip › Data/地级市Z_F工作报告.zip!/╜¡╦╒/┴1⁄4╘╞╕█╩╨/└┤╫╘╣1⁄2╓┌║┼í╛┬φ┐╦╩2╛▌═°í┐_2015.docx]

各位代表：

现在，我代表市人民政府，向大会报告工作，请予审议，并请各位政协委员提出意见。

2014年工作回顾

过去一年，我们在省委、省政府和市委的坚强领导下，团结和依靠全市人民，积极应对复杂多变的宏观经济形势，坚持以改革创新促发展，以扩大开放求突破，在困难和挑战较多的情况下，较好地完成了市十三届人大三次会议确定的目标任务。预计全年实现地区生产总值1920亿元，增长10.3%；规模以上工业增加值989.8亿元，增长12.4%；一般公共预算收入261.8亿元，增长12.2%；全社会固定资产投资、社会消费品零售总额、外贸进出口总额分别增长23.6%、13%、20.8%；居民消费价格基本稳定，城镇和农村居民人均可支配收入分别增长10.3%、12.3%。十大民生工程和百件惠民实事顺利实施，一批社会关注、群众关切的事项得到解决，在加快改革发展过程中群众得到实惠。

一年来，我们主要做了以下工作：

一、抢抓国家战略机遇，一带一路交汇点建设取得突破

区域合作开创新局面。在两国元首共同见证下，中国和哈萨克斯坦共和国国际物流合作基地一期工程建成启用，成为丝绸之路经济带和21世纪海上丝绸之路首个经贸合作实体项目。全面深化对外合作，与吉尔吉斯共和国首都比什凯克结为友好城市，与立陶宛共和国交通部签订共建海陆链网合作协议，与巴西淡水河谷公司确立友好合作关系，中韩陆海联运列入全国试点。一带一路农业国际合作示范区落户我市。成功举办首届中国（连云港）丝绸之路国际物流博览会。连云港被国家一带一路战略规划确定为新亚欧大陆桥经济走廊首要节点城市、中哈物流中转基地和上海合作组织出海基地。

重大建设加快推进。港口完成固定资产投资45亿元，连云港区3个通用泊位完工，徐圩港区4个泊位全面建成，赣榆港区一期液体化工泊位、燕尾港区2个5万吨级泊位投入试运营，灌河港区两个2万吨级通用泊位建成运营，新增吞吐能力1600万吨。新国际客运站投入使用。集疏运体系不断完善，旗台作业区铁路专用线建成，北疏港高速公路隧道贯通。连盐铁路、连淮扬镇铁路开工建设，临海高等级公路建成通车，新机场选址获国家批复。田湾核电站3号机组完成穹顶吊装，500千伏南翼输变电工程获得核准。

开放水平不断提高。在长三角、珠三角和中西部等地务实开展招商引资活动，全年引进内联到位资金948.7亿元，增长23.6%；实际利用外资增长18.7%，增幅全省第二。外贸进出口总额80.2亿美元，增长20.8%，增幅全省第一，其中对俄罗斯、中亚五国出口分别增长26.8%和67.3%。新核准境外投资项目21个。全市开发园区完成业务总收入5900亿元，增长14%。获批全国首批进境粮食指定口岸。获准实施启运港退税政策。开展口岸查验三互、三个一改革。全面启动长三角通关一体化及苏北五市检验检疫一体化工作，徐圩港区实现口岸临时开放。

二、坚持扩量提质并重，产业转型升级步伐加快

工业经济稳中向好。装备制造、石化产业产值突破千亿，冶金产业实现产值800亿元，新医药、新材料、新能源产业实现产值1300亿元。宏创药业、亚邦产业园等124个亿元以上工业项目开工建设，珠江钢管、虹港石化、益海特种油等108个亿元以上工业项目建成投产，新增规模以上企业245家，销售收入过百亿企业达5家。实施亿元以上技改项目117个，完成工业技改投资605亿元，工业设备投入增速全省第一。逐步淘汰落后产能，单位GDP能耗同比下降3%。

重点服务业快速发展。69个重点项目完成投资118.5亿元。物流产业实现产值700亿元。新增各类融资380亿元，其中直接融资162亿元，太平洋石英成功上市，全市上市公司达7家，10家企业在新三板和区域性股权交易中心挂牌。新增国家4A级旅游景区3个，花果山获批国家地质公园，云台山风景名胜区荣膺中国十佳海洋旅游目的地。

现代农业稳步发展。新增设施农业11.5万亩、适度规模经营32.7万亩。新批省级农业园区、出口示范区各2家，出口示范基地总数全省第一，农产品出口4.5亿美元。新建放心农资直营店110家。全面完成粮食危仓老库改造任务。高标准农田比重苏北第一，主要粮食作物生产基本实现机械化。粮食生产实现十二连丰。

创新能力得到提升。全市科技进步贡献率达51%，全社会研发支出占地区生产总值比重达1.7%，新增国家级高新技术企业38家。市科创城被确定为国家级科技企业孵化器，中科院能动中心开始气化炉试验。医药产业创新能力全国领先，在全国新批的5个创新药中恒瑞、豪森各获1个，豪森荣获国家科技进步二等奖和中国专利金奖。创成国家级海外智力工作基地。全年引进高层次人才1020名。

三、统筹城乡规划建设，新型城镇化稳步推进

城市布局进一步优化。顺利完成赣榆、海州区划调整，行政区划与功能组团关系逐步理顺。全市城镇化率提高到59%。

主城区功能日益完善。城区基础设施配套得到加强，一批城市综合体和历史文化街区建成营业。交通路网得到优化，海滨大道、环云台山大道基本贯通，打通科苑路、运河路等5条断头路，完成人民路、大港路等14条主次干道改造。市区新辟公交线路7条，优化调整12条。完成旧城改造550万平方米，拆除违法建筑6.6万平方米。

城乡建设扎实推进。三县县城承载能力加速提升，东海滨河新区功能快速完善，灌云东城区配套建设加快，灌南硕项湖片区初具功能。全市镇村规划实现全覆盖，重点中心镇加快建设，示范镇村建设投资达34亿元，温泉镇、桃林镇创成国家级宜居小镇。区域供水、污水处理及镇村垃圾转运等基础配套逐步完善，建成农村公路600公里、桥梁88座。

生态建设力度加大。水污染防治、大气污染治理等生态环境整治成效显著，超额完成省定PM2.5削减任务。强力推进化工园区专项整治，关闭化工企业34家、责令整治187家。整治燃煤小锅炉103台，淘汰老旧机动车1.1万辆。秸秆综禁工作扎实有效。积极开展海洋生态修复工程，秦山岛一期工程完工，港口航道生态修复全面实施。市级森林防火指挥中心建成投用。绿化造林28.8万亩，林木覆盖率达28.6%。通过国家节水型城市考核验收。东海、赣榆建成省级生态县区。

四、扎实推进民生幸福工程，群众生活一年更比一年好

饮水安全得到保障。群众的饮水安全一直是政府牵挂的大事。经过三年不懈努力，投入资金20亿元，开挖蔷薇湖，新辟沭新渠，引得活水来，城区饮用水水质得到明显改善，百万居民喝上了更加干净的水；在农村偏僻地区实施自来水进村入户工程，建管网，净水源，去年又有43万农民喝上了安全的自来水。

居住环境得到改善。投入2.1亿元，整治市区14个低洼易涝片区、1167共120公里背街小巷，去年雨季没有再发生大面积积水，30万群众直接受益。改造老旧小区60个。大力实施棚户区改造，完成建筑面积48万平方米，5300户居民喜迁新居。初步完成1800个村庄环境综合整治，农村居民生产生活环境不断改善。

富民工程加快实施。加大就业创业扶持力度，完成各类职业技能和创业培训29.3万人次，新增城镇就业7.6万人、农村劳动力转移就业3.6万人，下岗失业人员再就业1.9万人，城镇登记失业率2.4%。促进重点群体就业，高校毕业生总体就业率稳定在90%以上。加强家庭困难人员就业援助，城镇零就业家庭和农村零转移家庭保持动态为零。推进创业型城市建设，强化小微企业政策扶持，全市新设立企业数、注册资本金分别增长18.6%和128.2%。深入开展石梁河库区等片区集中扶贫开发，100个村、12万人实现脱贫。

社会保障不断强化。城镇职工五项保险参保率稳定在95%以上，城镇居民医保覆盖率达96%，城镇职工、城乡居民合规住院费用报销比例达80%和70%。实现企业退休人员养老金十连调和城乡居民保险基础养老金三连调，新增失地农民全部进保。新开工各类保障性住房5738套，首个公租房小区竣工。养老服务体系不断完善，建成区域性养老服务中心49个，城市社区居家养老服务中心实现全覆盖。全面落实城乡低保、困难居民医疗救助自然增长机制，近7万名残疾人得到相应保障。

社会事业加快发展。改造中小学校舍45万平方米，师专二附小新校区、市公共卫生中心等重点公共服务设施建成投用。教育现代化建设苏北领先，东海、灌云成为国家义务教育发展均衡县。代表江苏省参赛的新海实验中学荣获2014中国汉字听写大会总冠军。县级公立医院均实行药品零差率销售，所有政府办基层医疗卫生机构实施基本药物制度。免费婚检率达81%。文化事业繁荣发展，成功举办连云港之夏、第八届中国曲艺节等系列活动，20件文艺作品获省级以上表彰，组织开展文化惠民活动2.26万场次，我市成为省级公共文化服务标准化试点地区。市区10分钟体育健身圈基本建成，我市运动员在青奥会、省运会上取得好成绩。

社会治理不断完善。村、居两委完成换届选举，创成省级民主法治示范村（社区）443个。建成政社互动试点社区40个，乡镇街道社会管理服务中心实现全覆盖。加强平安港城建设，刑事发案总量同比下降4.1%、破案总量同比上升3.7%。以五大专项行动为抓手，积极推进信访百案化解，持续开展消防、道路交通、校园安全等专项整治，安全生产形势总体平稳，社会秩序和谐稳定。强化食品药品安全监管，查办了一批重大制售假冒伪劣食品药品案件。查处价格违法行为91起。推进公民道德建设，赣榆区法院姜霜菊被中宣部评为全国最美基层干部，4人荣登中国好人榜。推进军地融合发展，创成省级双拥模范县区5个，灌云县开山岛夫妻哨王继才夫妇被授予全国时代楷模。民族宗教、气象、防震减灾、地方志、外事、侨务和对台工作得到加强，红十字、慈善、老龄和关心下一代等各项事业全面发展。

在统筹做好各项工作过程中，坚持以全面深化改革为引领，着力突破重点领域和关键环节。行政审批制度改革不断深化，市级审批项目由545项减至97项，基本建设审批项目用时由500天减至100天以内，审批事项集中率由57%提高到100%。财税改革不断深化，初步建立政府全口径预算制度，政府性债务管理进一步规范。金融改革创新力度加大，东海农村信用合作联社改制为农村商业银行，4家金融机构开展中小微企业融资周转方式试点。农村综合改革扎实推进，镇村土地确权登记颁证工作有序展开，在全省率先建成县乡两级农村产权交易市场体系。坚持以开展党的群众路线教育实践活动为契机，切实加强政府自身建设。严格落实中央八项规定和省市有关要求，认真抓好整改，注重建章立制，群众反映强烈的四风问题得到较好解决。法治政府建设阶段性目标通过省考核。受理软环境和行政效能投诉900件、办结率达96.9%，12345服务平台受理案件31万件、处置率达98.7%。自觉接受人大及其常委会的法律监督、工作监督和人民政协的民主监督，办结人大代表建议145件、政协委员提案320件。政务微博微信连云港发布上线运行。严格落实党风廉政建设责任制，强化审计监督和行政监察。完成机关办公用房、公务用车清理，公车购置量下降72%。

各位代表，过去的一年，全市上下负重前行、砥砺奋进，取得的成绩实属不易。这是省委、省政府和市委坚强领导的结果，是市人大、市政协鼎力支持的结果，是港城人民团结拼搏的结果。我代表市人民政府，向全市人民致以崇高敬意！向各位人大代表、政协委员，向各民主党派、无党派人士，向各人民团体，向驻连部队、武警官兵、公安干警，表示衷心感谢！向所有关心支持连云港发展的老干部、老同志、社会各界人士和海内外友人，表示诚挚谢意！

回顾过去的一年，我们也清醒地看到，区域竞争压力不断加大，加快发展的任务十分艰巨；低附加值产业比重偏高，重大项目招引不够理想；资源环境约束日益趋紧，部分企业生产经营困难；社会民生领域欠账依然较多，与人民群众的期盼还有差距；中梗阻现象在一些部门还不同程度存在，软环境建设需要常抓不懈；社会公共管理还存在薄弱环节，政府自身建设需要不断加强。我们将正视这些问题，采取有力措施，切实加以解决。

2015年主要工作任务

今年是全面深化改革的关键之年，是全面推进依法治国的开局之年，也是十二五规划的收官之年。我们要深刻理解经济发展新常态的特征和要求，把握好改革发展稳定大局，坚持从实际出发，科学谋划港城发展。

今年政府工作的总体要求是：全面贯彻落实党的十八大和十八届三中、四中全会精神，按照习近平总书记视察江苏重要讲话要求，认真落实省、市委工作部署，积极适应经济发展新常态，抓住用好一带一路交汇点建设重大机遇，坚持稳中求进总基调，以全面建成小康社会为目标，以加快产业发展为主题，全面深化改革，扩大对外开放，推进依法治市，统筹做好稳增长、调结构、抓创新、重生态、惠民生、防风险各项工作，全力推动连云港经济社会发展迈上新台阶。

主要预期目标是：地区生产总值增长11%左右，公共财政预算收入、城乡居民收入与经济发展同步增长，规模以上工业增加值增长14%左右，规模以上固定资产投资增长21%左右，社会消费品零售总额增长13%，外贸进出口总额增长12%，直接利用外资12.5亿美元，港口吞吐量增长10%，集装箱运量增长12%，居民消费价格指数不高于省控线，节能减排完成省定目标。

围绕以上目标要求，重点做好以下工作：

一、突出一带一路交汇点建设，全力增创开发开放新优势

抓住用好国家重大战略机遇，主动出击、务实作为，放大向东开放优势，做足向西开放文章，不断提升海陆双向开放层次。

加快组合大港建设。开工建设30万吨级航道二期工程，基本建成徐圩防波堤东堤、赣榆港区10万吨级航道、灌河5万吨级泊位。完善集疏运体系，加快连盐铁路徐圩、赣榆港区支线建设，建成北疏港高速公路，打通徐圩港区对外连接公路。科学规划内河码头布局，推进灌河航道整治工程，构建海河联运航道网络，实现与京杭运河三级航道贯通。全面落实启运港退税政策，推进国家集装箱海铁联运物联网示范工程，加快集装箱码头服务标准化建设。深化三互、三个一改革和口岸信息化建设，突出大陆桥运输优势和桥头堡功能，开行连新亚、连新欧班列，充分发挥港口在一带一路交汇点建设中的核心资源配置作用。

构建区域合作平台。深化与哈萨克斯坦共和国战略合作，开工建设中哈物流基地二期工程。拓展与立陶宛共和国、乌兹别克斯坦共和国、吉尔吉斯共和国等国家合作，促成上合组织国家在连设立办事机构。推进上合组织国际物流园、连云港国际农业示范区和中捷水晶文化产业合作园建设。建立与欧亚资源集团、巴西淡水河谷公司等跨国企业战略合作，共建国际矿产交易中心。打造连博会、国际港航论坛等展会品牌。深化与中西部地区合作，启动一带一路国际经贸合作区建设，实施多式联运中心、大宗资源采购中心等项目，促进港口共用、园区共建、政策共享，国家东中西区域合作示范区建设初见成效。

强化重大基础设施支撑。204国道绕城段全面开工建设，310国道市区段基本建成，徐新公路全线贯通，海滨大道全面建成通车。加快电网和特高压工程建设，开工500千伏南翼输变电、田湾核电站二期送出工程，建成11个110千伏及以上输变电工程，推进抽水蓄能电站建设，争取田湾核电三期工程通过核准。加快新机场前期工作。推进连盐、连淮扬镇铁路建设，开工建设青连铁路，推进陇海客运专线徐连段列入国家十三五规划，并尽早开工建设，全力推动连云港迈入高铁时代。

大力发展开放型经济。推动开发园区二次创业，加快特色发展，引领转型升级，各类园区实际利用外资、工业增加值占全市比重分别达60%和80%以上。推进宋跳工业园、新海新区、大浦工业园融合发展，着力打造国家级高新区。板桥工业园融入徐圩新区。突出重点区域，围绕重点产业，紧盯重点企业，开展精准招商。按照谁招商、谁受益原则，推动招引项目跨区域集聚。优化园区服务，建立健全帮办、代办制度，实现园内事园内办结。整合出口加工区、保税物流中心等海关特殊监管区，综合保税区、自由贸易港区申报实现突破。开展跨境双向人民币资金池试点，推进跨境电子商务发展。加快外经贸综合服务平台建设，打造区域性进口消费品市场和物流配送中心。实施跨国经营企业培育计划，加快走出去步伐，提升企业国际化水平。

二、突出产业强市战略导向，加快结构优化调整

强化创新引领，坚持先进制造业和现代服务业双轮驱动，做强支柱产业，壮大主导产业，全面加快新型工业化进程。

加速工业经济扩量提质。实施工业突破行动，着力抓好200个市级重点工业项目建设，确保100个新增长点项目达产达效，完成规模以上工业投资1270亿元、增长22%，净增规模以上企业100家。大力发展基础性、基地型临港产业，5000万吨国家级石化产业基地规划获批，盛虹石化项目获得核准，加快实施斯尔邦石化、天元锰业新材料等项目，石化、冶金产业产值分别达到1100亿元、900亿元，装备制造业产值达到1350亿元。推动新医药、新材料、新能源产业集群发展，产值达到1500亿元。实施日出东方太阳能等100个重点技改项目，完成规模以上技改投资750亿元。

大力发展服务业。着力抓好服务业集聚区和重点项目建设，服务业增加值增长12%。优化物流园区规划，完善城市配送体系，培育发展新型物流企业。不断壮大金融产业，大力引进各类金融机构，争取组建江苏沿海开发银行。创新金融产品服务模式，推动金融与实体经济融合发展，新增各类融资400亿元以上。重视会展经济发展，提升商务会展专业化水平。推进白虎山批发市场改造，加快万达广场等重大商贸设施建设，提升城市商业服务水平。理顺旅游管理体制机制，整合核心旅游资源，加大保护与开发力度，创成花果山国家5A级景区。全年游客接待量、旅游总收入分别增长10%、12%。

拓展产业发展空间。加快海洋强市建设，壮大蓝色经济，着力突破海洋高新技术产业，积极发展海水淡化、海洋生物、船舶与海工装备等产业，全市海洋经济总产值突破500亿元。做大文化产业，重点发展西游记文化、淮盐文化、水晶文化等地方特色产业，进一步提高文化产业占地区生产总值的比重。把握产业发展新趋势，加快引进新业态、新模式，大力培育电子商务、物联网、云计算、服务外包等产业。做强建筑产业，出台扶持政策，鼓励企业提档升级、创建品牌，建筑业总产值达到700亿元。

深入实施科技创新工程。加快国家创新型试点城市建设，完成国家级高新区创建。推进产学研用协同创新，积极申报国家重大科技基础设施项目，新增专利授权3000件，全社会研发支出占地区生产总值比重提升到1.85%。实施科技企业培育工程，加快孵化器、加速器和中试基地建设，推动大型科学仪器设备共享共用。推动创新资源向企业集聚，新增企业研发机构30个、国家级博士后科研工作站1家，新培育高新技术企业30家，高新技术产业产值突破2000亿元。推进质量强市，新创省级以上名牌产品10个、国家地理标志保护产品2个。加强人才培育和招引，创造条件吸引行业领军人物、拔尖人才落户港城，引进高层次人才1100名以上。

完善企业服务体系。开展企业服务年活动，健全领导挂钩联系企业、难题集中会办机制，切实帮助企业解决困难。开展企业减负专项行动，实施涉企收费目录清单管理。组建市级中小企业服务中心，建成中小微企业公共服务平台。推进小微企业转贷方式创新，设立中小企业融资超市、工业过桥资金、企业融资信息平台，推广苏科贷等科技金融服务产品，努力破解中小微企业融资难题。加强各类商会和中介组织建设，推动建立产业联盟和行业协会，充分发挥其信息咨询服务、行业协同发展、维护企业合法权益的重要作用。

三、突出组团融合发展，进一步增强城市集聚辐射功能

强化规划引领，坚持集聚集约发展，把握好城市建设重点和节奏，加快国际性海滨城市建设。

优化城市规划布局。按照建设一带一路交汇点的发展定位，港口、产业、城市融合发展的思路，经济社会发展、土地利用、城乡建设、生态环境保护等规划多规合一的要求，开展城市总体规划修编，完成中期评估。加快推进重点片区和地块控制性规划编制工作，优化东部城区空间布局，统筹推进连云区和连云新城建设，重点完善商务办公、港航物流、旅游休闲功能，打造区域性国际商务中心。海州城区合理控制开发规模和时序进度，重点实施成片旧城改造、重点街区打造，着力提升主城区形象内涵。赣榆城区加快融入主城区，逐步实现功能互补、设施对接、一体发展。

提升城区功能品质。优化城市路网，建成环云台山大道等一批主次干道，打通人民东路、建设西路等9条断头路，推进秦东门大街、镇海路等66条破损道路整修，启动东盐河慢行系统建设，新辟和优化公交线路10条，更新公交车100辆，城市公共交通分担率提高到23%。完善基础设施，新增市政管网60公里，改造老旧管网50公里。刘湾垃圾填埋场建成投用，餐厨垃圾处理工程主体完工。实施市区防洪排涝提升工程、病险塘坝整治工程。提升公共配套，推进科技创业城、新世界文化城、市第一人民医院新海新区医院等重点项目建设。完成旧城改造550万平方米，整治背街小巷600条共60公里，基本完成低洼易涝片区改造，提升城市人居环境。

切实加强城市管理。坚决查处违法建设，全面改善交通拥堵、抛撒滴漏、占道经营等状况。提高道路、广场、绿地管养水平。绘制全覆盖的地下管网分布图，推行建筑工地、地面广场等实时监控管理新模式。完善物业管理体制，着力解决物业管理中的突出问题，改造老旧和无物管小区70个，全市物业管理覆盖率达87%。推进城市数字化、精细化管理，健全快速反应和长效管理机制，着力营造整洁、有序、文明的城市环境。

四、突出改革创新，充分激发经济社会发展活力

坚持问题导向，勇于创新实践，着力解决发展中的体制机制障碍，推动各项改革尽快落地见效。

深化行政体制改革。全面推行权力清单制度，完善权力运行和监督机制，让权力在阳光下运行。健全行政审批和公共服务网上运行机制，推进审批项目、服务事项和中介机构向政务服务中心集中，打造一站式服务平台。稳步推进政府机构改革，全面推行绩效管理制度。加快事业单位分类改革。完成公车改革。加大政府购买服务力度，在公共服务领域引入市场竞争机制。创新供水、供热运营机制，探索组建政府主导的公用事业专业化公司。推进扩权强镇，抓好中心镇改革试点，激活县区和重点区域发展潜能。

深化经济社会体制改革。实施新一轮市、区财政管理体制改革，完善全口径政府预算体系，全面推进三公经费预决算公开。做好机关事业单位养老保险制度改革。加快国资国企管理体制改革，推进国有企业分级分类管理和功能性重组，完善法人治理结构和现代企业制度，推动发展混合所有制经济。上半年完成港口集团改革。加强政府性债务管理，推动投融资平台转型发展，提高经营管理水平和风险防范能力。组建工业投资集团，发挥国有资本杠杆作用，促进工业经济加快发展。构建多层次资本市场，推动更多企业上市和在新三板挂牌。组建市属金融控股公司，推进地方金融资源整合，优化金融生态环境。启动不动产登记制度改革。创新社区减负增效和社会组织管理制度。加快户籍制度改革，制定差别化落户政策。加强经济社会发展研究，以改革创新的精神科学编制十三五规划。

深化农村综合改革。坚持土地公有制性质不改变、耕地红线不突破、农民利益不受损三条底线，推进以产权制度改革为核心的农村综合改革，赋予农民更多财产权利。健全农村产权权益实现机制，基本完成耕地确权登记颁证，推进集体建设用地确权登记，开展农民住房确权登记试点，建立县、乡两级农村产权交易综合平台，促进城乡要素平等交换和公共资源均衡配置。完善土地、滩涂流转制度，新增土地流转20万亩。推进小型农田水利管理体制改革，健全长效管护机制。

五、突出城乡统筹，推动农业农村发展迈上新台阶

围绕农业强、农村美、农民富目标，重点推进农业现代化、城乡一体化发展，实施精准扶贫，打好全面小康建设攻坚战。

加快转变农业发展方式。稳定粮食生产，重点打造80个万亩示范区，新增设施农业8万亩，高标准农田比重提高到50%。培育新型农业经营主体，新增家庭农场、农民专业合作社各200家，新增省级以上龙头企业7家、规模以上龙头企业20家。基本建成高公岛国家一级渔港。实施农业国际合作示范区规划，重点建设东海、云台等5个示范片区，食品工业、农业装备制造等6个基地，实现农产品出口4.8亿美元。推动农业生产全程机械化，开展粮食烘干试点。全面推广农业园区、粮食生产功能区标准化生产模式，完善市级农业标准信息服务平台。加强农技服务体系建设，促进农业科技进村入户。

改善农村生产生活条件。编制新型城镇化与城乡发展一体化规划，进一步构建完善的城镇体系，推动小城镇逐步向小城市过渡，提升县城、重点中心镇承载发展能力。加快农民集中居住区建设，发展布局合理、设施配套、有利生产、方便生活的新型农村社区。加快建设美丽乡村，整合资源、集中力量，全面完成村庄环境整治三年目标任务。完成农村改厕3万座。推进区域供水工程建设，解决农村60万人饮水安全问题，区域供水覆盖率提高到85%。建成农村公路300公里，改造公路桥梁30座。实施水美乡村工程，疏浚县乡河道1061公里，整治村庄河塘1067个。发展放心农资直营店80家、放心粮油店100家。

大力开展扶贫攻坚。突出石梁河库区和新沂河两岸两大重点片区开发，实现14万低收入人口、89个经济薄弱村脱贫。加大贫困地区投入力度，重点实施一批交通、水利等基础工程。完成2000户农村危房改造，实现260户渔民上岸安居。全面落实强农惠农政策，大力发展镇村经济。拓宽村集体增收渠道，加强村级减负增效治理，严控薄弱村新增债务。公开公正进行低收入农户认定工作。发挥中小企业园和扶贫产业园载体作用，健全薄弱村、贫困户稳定脱贫保障机制，确保全面完成新一轮扶贫开发任务。

六、突出生态建设，保护好连云港的自然之美

生态文明建设关系百姓生活，关乎城市未来。必须下决心用硬措施完成硬任务，让港城的山更绿、水更清、天更蓝。

坚决抓好节能减排。积极推进城区和园区集中供热，加快燃煤小锅炉整治工作，实施重点节能改造项目35个，力争节约15万吨标煤。完成32个工业和50个农业减排项目，实现十二五减排目标。深入推进化工园区整治，加强园区污水处理、危险废物处置等环境基础设施建设，加快实施园区敏感目标搬迁，确保通过省级考核验收。加大污水处理厂和管网等配套设施建设力度，城市生活污水处理率不低于80%。启动德邦化工、连云港碱厂搬迁工作。建立落后产能淘汰退出制度，坚决关停并转高耗能、重污染企业。

着力抓好大气污染防治。深入开展扬尘污染控制，抓好港口散货堆场覆盖，严格建设施工、道路运输扬尘管控，城市建成区主要车行道机扫率达75%以上。基本完成黄标车和老旧机动车淘汰任务。燃煤电厂实施超低排放，推动钢铁、水泥、化工类企业实施提标改造。重抓秸秆禁烧与综合利用，巩固常态化管理机制。健全监测预警应急体系，有效防范和应对重污染天气，力争空气优良天数达255天以上。

持续改善水环境质量。完成蔷薇湖备用水源建设，全面建成饮用水输水工程，确保沭新渠稳定供水。加强饮用水源沿线污染源整治，加快提升沿线区域污水处理能力，确保东海-海州尾水通道工程投入使用。开展排淡河、玉带河、西盐河等城市河道整治，基本消除黑臭现象。加强农业面源污染和近海海洋环境污染治理，稳步提升地表水体水质达标率。

扎实推进生态建设。力争建成全国绿化模范城，东海县、赣榆区达到国家级生态县区标准，其他县区达到省级生态县区标准。坚决遏制私挖乱采行为，抓好废弃塘口整治复绿。新增造林12万亩，林木覆盖率达到29.1%。严格耕地保护和生态红线制度，全面落实土地节约集约利用措施，加强生态湿地保护，实施水土保持和海洋生态修复工程，推进海岛整治修复和综合开发利用，加快建设海州湾国家级海洋公园。

七、突出惠民利民，致力增进民生福祉

扎实推进十大民生工程，办好百件惠民实事，以更大力度提供更多更好的公共服务，不断提高人民群众的满意度和幸福感。

切实增加居民收入。大力推进全民创业，制定出台鼓励创业政策，完善扶持创业服务体系，新增私营企业9500户、个体工商户2万户。启动实施大学生创业引领计划，扶持大学生创业1000人。完成各类职业技能培训25万人次，新增城镇就业8万人、转移农村劳动力3万人，城镇登记失业率控制在3%以内。政府购买公益性岗位2000个，援助困难人员就业，保持城镇零就业家庭和农村零转移家庭动态为零。健全企业工资收入分配制度，完善工资决定和正常增长机制。

完善社会保障体系。巩固扩大城镇职工和城乡居民社会保险覆盖范围。城镇职工、城镇居民医保和农村居民新型农村合作医疗合规住院医疗费用报销比例稳定在80%、70%和75%以上。征地补偿标准动态调整，逐步提高被征地农民基本生活保障标准。住房公积金覆盖率达68%以上。加大住房保障力度，推进公租房与廉租房并轨运行，新开工各类保障性住房2930套，建成各类保障性住房3790套，城镇住房困难家庭应保尽保。完善养老服务体系，建成区域性养老服务中心21个，全面完成农村养老机构提档升级任务，每千名老人拥有养老床位增至31张。关爱农村留守妇女儿童，建立困境儿童救助保护制度。推进残疾人社会保障体系建设。提高社会救助规范化水平，落实低保标准、优抚增长机制，积极推动基层慈善组织网络建设。

加快发展社会事业。以省教育现代化市级示范区建设为抓手，统筹推进学前教育改革发展、义务教育均衡发展，加快职业教育创新发展、高等教育特色发展。创建省优质幼儿园16所，改造中小学校舍45万平方米，义务教育学校现代化建设达省定标准比例50%。支持连云港师专申建海州师范学院、淮海工学院恢复淮海大学。完善基本医疗卫生体系，加强基层医疗卫生机构建设，加快构建分级诊疗体系，推进城市公立医院全面实施药品零差率销售，基层医疗卫生机构、村卫生室全部实施基本药物制度。免费孕前优生健康检查覆盖率达90%以上。大力倡导全民阅读，实施全民科学素质提升工程。建设全媒体城市综合信息服务平台，启用广播影视文化产业城。市档案馆新馆主体完工，市民政公共服务中心建成使用。开展省公共文化服务标准化创建工作，推进基层服务设施提档升级，力争万人拥有公共文化设施1200平方米。建设市全民健身中心，人均体育设施达2平方米。

加强和创新社会治理。全面深化平安连云港建设，力争在全省率先建成和谐稳定示范区。扎实开展安全生产行政执法监督年活动，强化车辆超限超载治理、火灾隐患整改、建筑施工监管等工作，确保安全生产形势持续稳定。严厉打击非法集资和金融诈骗活动。强化校园周边环境综合治理和校车安全管理。推进食品药品放心工程，切实保障舌尖上的安全。全面推行阳光信访，推动信访纳入法治化轨道，妥善解决征地拆迁、房地产等领域历史遗留问题。建立健全社会信用体系，推进信用信息互联共享。全面完成六五普法，省级民主法治示范村（社区）覆盖率达30%。深入推进政社互动和三社联动，加强互联社区建设。整合应急资源，全面提升综合应急能力。深入开展群众性精神文明建设，以先进模范引领社会风尚。充分利用本土红色文化，广泛开展爱国主义教育，大力推进军地融合发展，争创全国双拥模范城七连冠。

努力建设法治政府和服务型政府

以改革的精神、创新的思维、法治的方式，推进政府治理体系和治理能力现代化建设。

全面推进依法行政。健全依法决策机制，规范重大行政决策程序，落实公众参与、专家论证、风险评估、合法性审查、集体决定等法定程序，积极推行政府法律顾问制度，建立重大决策终身责任追究制度和责任倒查机制。深化行政执法体制改革，完善市县两级行政执法管理，推动行政执法重心下移、力量下沉。规范公正文明执法，建立健全行政裁量权基准制度，确保依法办事、程序正当。自觉接受人大的法律监督、工作监督和政协的民主监督，主动接受社会公众的舆论监督，坚持定期向人大常委会报告工作，向政协通报情况，认真听取各民主党派、工商联、无党派人士和人民团体的意见。

大力提高行政效能。全面落实中央八项规定和省、市委的各项规定，巩固扩大党的群众路线教育实践活动成果。以群众满意为标准，持续开展庸懒散、不作为、乱作为专项治理，全面推进软环境建设，切实改进机关作风、提高服务效能。严格落实岗位责任制、服务承诺制、首问负责制、限时办结制，切实提高行政执行力。全面推进政务公开，完善四级政务服务体系，加强互联网政务信息服务平台和便民服务平台建设，健全连云港发布运行机制，实现决策、执行、管理、服务、结果五公开。正确处理政府与市场关系，推进政府职能向提供公共服务、营造公平市场环境转变，更好地服务民生、助推发展。

切实加强廉政建设。严格财政资金、国有资产、公共工程建设等监管，强化政府行政监察和审计监督，从源头上预防和减少腐败现象发生。坚持以零容忍态度惩治腐败，严肃查处各类违法违纪案件，着力解决损害群众利益的突出问题。坚持艰苦奋斗，厉行勤俭节约，反对铺张浪费，严格财务预算制度，严控三公经费和一般性支出，坚决遏制工程建设领域超预算现象。严格执行党风廉政建设责任制，持之以恒纠正四风，始终保持为民、务实、清廉的良好形象。

各位代表，连云港发展的历史机遇弥足珍贵，需要我们顺时而谋、乘势而上；肩负的使命艰巨光荣，需要我们心无旁骛、勇于担当；500万人民的热切期盼催人奋进，需要我们不负重托、奋发作为。让我们紧密团结在以习近平同志为总书记的党中央周围，在市委的正确领导下，戮力同心，脚踏实地，埋头苦干，为建设经济强、百姓富、环境美、社会文明程度高的新港城而努力奋斗！
